# Maternal Supplementation of Low Dose Fluoride Alleviates Adverse Perinatal Outcomes Following Exposure to Intrauterine Inflammation

**DOI:** 10.1038/s41598-018-38241-8

**Published:** 2019-02-22

**Authors:** Bei Jia, Lu Zong, Ji Yeon Lee, Jun Lei, Yan Zhu, Han Xie, Julia L. Clemens, Mia C. Feller, Quan Na, Jie Dong, Michael W. McLane, Kimberly Jones-Beatty, Irina Burd

**Affiliations:** 1 Integrated Research Center for Fetal Medicine, Division of Maternal Fetal Medicine, Department of Gynecology and Obstetrics, Johns Hopkins University, School of Medicine, Baltimore, MD USA; 2 The Center for Prenatal and Hereditary Disease Diagnosis, Department of Obstetrics and Gynecology, Nanfang Hospital, Southern Medical University, Guangzhou, 510515 China

## Abstract

Maternal periodontal disease has been linked to adverse pregnancy sequelae, including preterm birth (PTB); yet, root planing and scaling in pregnancy has not been associated with improved perinatal outcomes. Fluoride, a cariostatic agent, has been added to drinking water and dental products to prevent caries and improve dental health. The objective of this study was to explore the effects of fluoride supplementation using a mouse model of preterm birth and perinatal sequalae. Pregnant mice were fed low dose fluoride (LF^−^) or high dose fluoride (HF^−^) and given intrauterine injections of lipopolysaccharide (LPS) or phosphate-buffered saline (PBS). We found that LPS + LF^−^ significantly increased livebirths, pup survival, and litter size compared to LPS alone. Moreover, offspring from the LPS + LF^−^ group exhibited significantly improved neuromotor performance and more neurons compared to those from the LPS group. Additionally, LF^−^ treatment on human umbilical vein endothelial cells (HUVECs) increased cell viability and decreased oxidative stress after treatment with LPS. Collectively, our data demonstrates that maternal LF^−^ supplementation during pregnancy postpones the onset of PTB, acts to increase the liveborn rate and survival time of newborns, and reduces perinatal brain injury in cases of intrauterine inflammation.

## Introduction

Periodontal disease is a common problem worldwide and has been associated with an increased risk for many comorbidities, including hypertension^1^ and diabetes^2^, as well as adverse pregnancy outcomes, including preterm birth (PTB) and low birthweight of neonates^3,4^. Although this connection is known, interventions designed to improve periodontal disease, such as scaling and root planing, have not been useful in improving adverse pregnancy outcomes^5^. Additionally, studies including oral hygiene education, such as encouraging the use of mouthwash, tooth brushing, and flossing after meals, did not find a difference in the lengths of pregnancy gestation between women with and without periodontal disease^6,7^. Finally, treatment of periodontal disease that included antibiotics (metronidazole) did not have an effect on the PTB rate^8^. The mechanism behind the connection between PTB and periodontal disease remains unclear.

PTB, defined as delivery before 37 weeks gestation, represents a major public health challenge. Each year, approximately 15 million children are born prematurely around the globe^9^. PTB remains the leading cause of neonatal morbidity, constituting up to 70% of perinatal mortality worldwide^10,11^. Intrauterine inflammation (IUI) is strongly associated with PTB. Up to 40% of premature births exhibit clinical evidence of inflammation^12,13^, and exposure to IUI during prenatal development places offspring at risk for adverse neurodevelopmental outcomes, such as cerebral palsy, autism spectrum disorder, learning disabilities, and schizophrenia^14–16^.

The results of epidemiological, molecular, microbiological and animal-model studies also support a positive association between maternal periodontal disease (periodontitis) and PTB^17^. Periodontal disease is a gram-negative anaerobic infection of the mouth that affects up to 90% of the population^18^ and has been shown to be even more prevalent in the pregnant population^19^. Periodontal disease and PTB share many of the same common risk factors, including age, smoking habits, low socioeconomic status, and systemic health status. Also, previous research has suggested that maternal infections altering normal cytokine- and hormone-regulated gestation may result in preterm labor^20^. It has also been shown that the placental microbiome is similar to that of the oral cavity^21^. Periodontitis could act as a distant reservoir of both microbes and inflammatory mediators, which may influence pregnancy and contribute to the induction of PTB^22–25^. While intervention studies (e.g. scaling) have sought to determine the effect of periodontal treatment on reducing the PTB risk, the results remain controversial^17^.

Fluoride (F^−^) plays an important role in upholding good oral hygiene. It is classified as a cariostatic agent, which is a substance that blocks the formation of dental caries. Fluoridation of drinking water and dental products help to prevent dental cavities, indicating that the use of an appropriate amount of fluoride may improve oral health^26–28^. The National Academy of Medicine (NAM), previously known as the Institute of Medicine, recommends a daily intake of 3 mg of fluoride for women aged 14 to 50. The NAM also notes that most research indicates that a daily intake of 10 mg for 10 or more years is necessary to produce clinical signs of mild skeletal fluorosis; thus, the upper intake limit is regarded as 10 mg/day^29^. The Food and Drug Administration has also stated that 10 mg/day is the upper intake limit^29^. While fluoride has been linked to certain negative health outcomes, this has occurred only at extremely high doses^30^. The role of low dose fluoride (LF^−^) on pregnancy outcomes, although recommended by the NAM, has not been very well explored.

In the present study, we hypothesize that fluoride will decrease the rates of PTB caused by lipopolysaccharide (LPS) due to its beneficial oral health properties. Specifically, we aimed to explore the effects of fluoride supplementation at levels recommended by the NAM on obstetrical outcomes using a mouse model of IUI. Eventually, we propose that fluoride could be used as a prenatal supplement for pregnant women suffering from periodontal disease to decrease the risks of PTB.

## Results

### Maternal LF^−^ supplementation improved PTB rate prior to 24 hours (h) after intrauterine LPS injection

Timed-pregnant CD1 mice consumed water with 6 mg/L LF^−^ and 113 mg/L high dose fluoride (HF^−^) from embryonic day (E) 9 to postnatal day (PND) 19. On E17, an established mouse model of IUI was utilized as previously described^31–35^. Mice were monitored for PTB for 36 h after surgery. There were no preterm births among pregnant dams that received intrauterine phosphate-buffered saline (PBS) (n = 20) or intrauterine PBS with either LF^−^ (n = 17) or HF^−^ (n = 7) supplementation (Table 1). Pregnant dams given intrauterine LPS (n = 52) had a PTB rate of 50% prior to 24 h after injection. Treatment of LPS-exposed dams with maternal LF^−^ supplementation (n = 51) significantly reduced the PTB rate to 23.5% (Table 1, p < 0.01, Chi-squared test). In comparison, supplementation with HF^−^ significantly increased the PTB rate to 91.7% (n = 12) within 24 h of LPS-induced IUI (Table 1, p < 0.01, Chi-squared test). Dams in the LPS + HF^−^ group delivered prior to 24 h in 100% of the cases with no surviving pups. Most preterm deliveries in the LPS + LF^−^ group (43.1%) occurred at late preterm gestation between 24 and 36 h after injection compared to LPS group (23.1%) (Table 1, p < 0.05, Chi-squared test). There was no significant difference in the PTB rate between LPS (73.1%), LPS + LF^−^ (66.7%), and LPS + HF^−^ (91.7%) groups 36 h after intrauterine LPS injection (Table 1, p > 0.05, Chi-squared test).

**Table 1:** Low dose fluoride supplementation significantly decreases number of early preterm deliveries.

Group (Dams)	Preterm delivery prior to 24 h n (%)	Preterm delivery between 24 and 36 h n (%)	Total preterm delivery n (%)
PBS (20)	0 (0.0)	0 (0.0)	0 (0.0)
PBS + LF^−^ (17)	0 (0.0)	0 (0.0)	0 (0.0)
PBS + HF^−^ (7)	0 (0.0)	0 (0.0)	0 (0.0)
LPS (52)	26 (50.0)	12 (23.1)	38 (73.1)
LPS + LF^−^ (51)	12 (23.5)**	22 (43.1)*	34 (66.7)
LPS + HF^−^ (12)	11 (91.7)**	0 (0.0)	11 (91.7)

Dams with lipopolysaccharide (LPS)-induced intrauterine inflammation delivered preterm compared to dams given only phosphate-buffered saline (PBS). High dose fluoride (HF^−^, 113 mg/L) supplementation was found to significantly increase the number of early preterm deliveries within 24 hours (h) of injection. Supplementation with low dose fluoride (LF^−^, 6 mg/L) during pregnancy had a protective effect, and significantly fewer dams delivered at early preterm compared to LPS alone. Chi-squared test, *p < 0.05, **p < 0.01.

### Maternal LF^−^ supplementation increased the liveborn rate and the survival time of newborns in an intrauterine inflammatory environment

**Figure 1:**
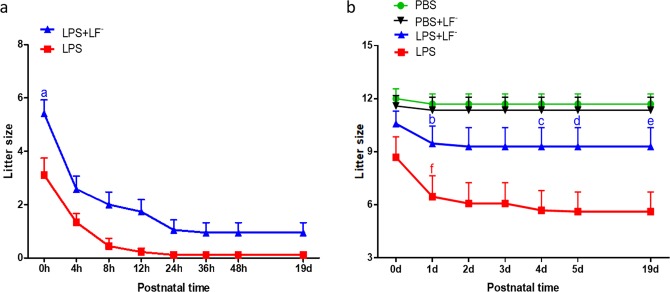
Survival time of pups increases after preterm birth and term birth with maternal low dose fluoride (LF^−^) supplementation. (**a**) In cases of preterm birth, maternal LF^−^ supplementation (n = 19) significantly increased litter size at 0 hours (h) in dams with lipopolysaccharide (LPS)-induced intrauterine inflammation compared to LPS alone (n = 9). Two-way analysis of variance (ANOVA), (**a**) LPS + LF^−^ (blue), p < 0.001, compared to LPS (red). Data are reported as mean ± SEM. (**b**) LF^−^ supplementation resulted in greater pup survival and litter size compared to LPS alone from postnatal day (PND)4 to PND19 in cases of term birth. Two-way ANOVA, (**c–e**) LPS + LF^−^ (blue), p < 0.05, compared to LPS (red). Term liveborn pups exposed to maternal LPS continued to die after birth, resulting in significantly reduced litter size from 1 day (**d**) to 19 d. Two-way ANOVA, (**f**) 1 d (red), p < 0.01 compared to 19 d (red). Maternal LF supplementation in dams exposed to LPS-induced intrauterine inflammation appears to prevent the death of pups following term birth. Two-way ANOVA, (**b**) 1 d (blue), p > 0.05 compared to 19 (**d**) (blue). Phosphate-buffered saline (PBS), n = 20; PBS + LF^−^, n = 17; LPS + LF^−^, n = 17; LPS, n = 13.

Maternal LF^−^ supplementation (55.9%) significantly increased the liveborn ratio by litter compared to LPS alone (23.7%) in cases of PTB (Table 2, p < 0.01, Fisher’s exact test). More pups born preterm in the LPS + LF^−^ group (17.6%) were able to survive until the end of the observation period (PND19) than with no fluoride supplementation (2.6%) (Table 2, p < 0.05, Fisher’s exact test). In cases of PTB, maternal supplementation with LF^−^ (n = 19) significantly increased litter size compared to LPS alone (n = 9) (Fig. 1a, p < 0.001, two-way ANOVA, LPS + LF^−^, 5.4 ± 0.5 compared to LPS, 3.1 ± 0.6). With maternal LF^−^ supplementation, more pups (0.9 ± 0.4) born preterm were able to survive without other medical treatment until PND19 than LPS alone (0.1 ± 0.1). Data are reported as mean ± SEM.

**Table 2:** Low dose fluoride supplementation significantly increases prevalence of live births and prolongs lifespan of pups born preterm.

Group (Dams with preterm birth)		LPS (38)	LPS + LF^−^ (34)
Litters with stillborn pups	n (%)	38 (100.0)	34 (100.0)
Litters with no liveborn pups	n (%)	29 (76.3)	15 (44.1)**
Litters with liveborn pups	n (%)	9 (23.7)	19 (55.9)**
Litters with survival pups on PND19	n (%)	1 (2.6)	6 (17.6)*

In groups of dams who had lipopolysaccharide (LPS)-induced intrauterine inflammation causing preterm birth, maternal low dose fluoride (LF^−^) supplementation had a significant positive effect. In the LF^−^ groups, significantly more litters had liveborn pups and fewer delivered only stillborn pups. Out of the litters with stillborn pups, significantly more litters had pups that were still alive on post-natal day (PND) 19. Fisher’s exact test, *p < 0.05, **p < 0.01 compared to LPS group.

In cases of term birth, maternal LF^−^ supplementation resulted in greater pup survival and litter size compared to LPS alone from PND4 to PND19 (Fig. 1b, p < 0.05, two-way ANOVA. PND4, LPS + LF^−^: 9.3 ± 1.1, LPS: 5.7 ± 1.1; PND5, LPS + LF^−^: 9.3 ± 1.1, LPS: 5.6 ± 1.1; PND19, LPS + LF^−^: 9.3 ± 1.1, LPS: 5.6 ± 1.1). Term liveborn pups exposed to maternal LPS continued to die after birth, resulting in decreased litter size from 6.5 ± 1.2 at PND1 to 5.6 ± 1.1 at PND19 (Fig. 1b, p < 0.01, two-way ANOVA). Maternal LF^−^ supplementation appeared to prevent the death of pups exposed to maternal LPS following term birth, with changes of litter size from 9.5 ± 1.0 at PND1 to 9.3 ± 1.0 PND19 (Fig. 1b, p > 0.05, two-way ANOVA). Data are reported as mean ± SEM.

### Maternal LF^−^ supplementation did not significantly affect maternal oral intake and pups’ birth weights

**Figure 2:**
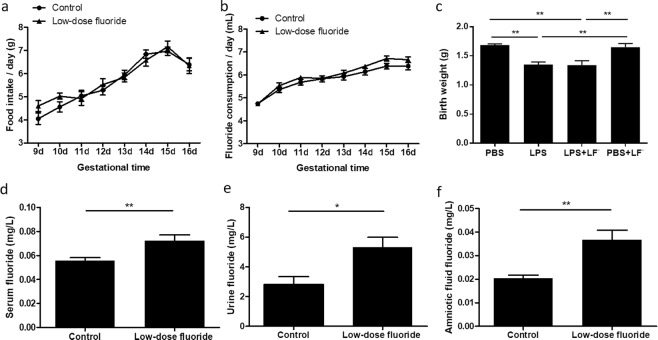
Maternal oral intake and pups’ birth weight were not affected by low dose fluoride (LF^−^) supplementation. However, maternal LF^−^ supplementation increased fluoride concentration in maternal serum, urine and amniotic fluid. There was no significant difference in either water consumption (**a**) or food intake (**b**) between control groups (n = 18) and groups that were supplemented with low dose fluoride (n = 20, two-way analysis of variance (ANOVA), p > 0.05), throughout gestation (d = day). Pups’ birth weight (**c**) was significantly decreased in pups from dams with lipopolysaccharide (LPS)-induced intrauterine inflammation (LPS, n = 7; LPS + LF^−^, n = 8), compared to phosphate-buffered saline (PBS) controls (PBS, n = 5; PBS + LF^−^, n = 7). However, there was no significant difference between LPS and LPS + LF^−^ groups. Data represent the mean ± SEM. One-way ANOVA with Bonferonni post hoc test, **p < 0.01. (**d**) There was a significant difference in levels of fluoride between control groups (n = 14) and groups that were supplemented with LF^−^ (n = 13) in maternal serum (Student’s t-test, **p < 0.01). (**e**) Maternal LF^−^ supplementation (n = 5) significantly increased the fluoride content in maternal urine compared to control group (n = 6) (Student’s t-test, *p < 0.05). (**f**) The results from amniotic fluid of LF^−^supplementation dams (n = 6) differ significantly from controls (n = 6) (Student’s t-test, **p < 0.01). Values are mean ± SEM.

There was no significant difference in either food intake or water consumption between the control group (n = 18) and the LF^−^ group (n = 20) (Fig. 2a,b, p > 0.05, Two-way ANOVA). There was a significant difference in birth weights between surgery controls (PBS, n = 5; PBS + LF^−^, n = 7) and maternal LPS-exposed groups with and without F^−^ supplementation (LPS + LF^−^, n = 8; LPS, n = 7) (Fig. 2c, p < 0.01, one-way ANOVA with Bonferonni post hoc test). However, there was not a significant difference between the LPS and LPS + LF^−^ groups.

### Maternal LF^−^ supplementation increased fluoride concentration in maternal serum, urine and amniotic fluid

Results of the fluoride determinations in E18 maternal serum, urine and amniotic fluid are presented in Fig. 2d–f. The highest fluoride concentrations were found in urine and the lowest in amniotic fluid. The mean (±SEM) amount of fluoride in the LF^−^ group was 0.072 ± 0.005 (serum, n = 13), 5.327 ± 0.662 (urine, n = 5) and 0.037 ± 0.004 (amniotic fluid, n = 6) mg/L, and in control group was 0.056 ± 0.003 (serum, n = 14), 2.792 ± 0.547 (urine, n = 6) and 0.020 ± 0.001 (amniotic fluid, n = 6) mg/L. Maternal LF^−^ supplementation significantly increased the fluoride content in maternal serum, urine and amniotic fluid compared to control group (Fig. 2d–f, p < 0.01 or p < 0.05, Student’s t-test).

### Maternal LF^−^ supplementation prevented adverse neuromotor outcomes in offspring exposed to IUI

**Figure 3:**
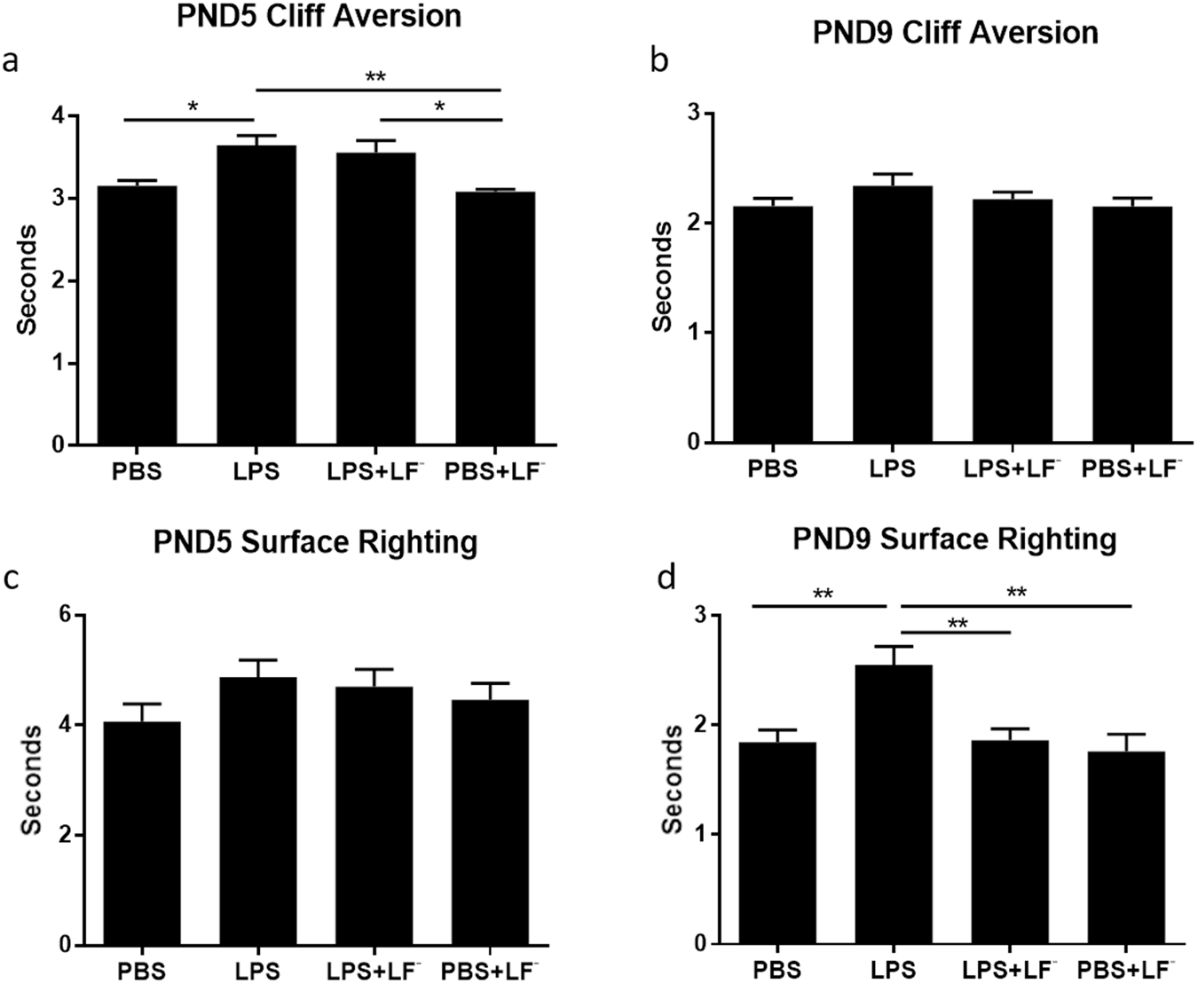
Neurodevelopmental testing is improved in groups with low dose fluoride (LF^−^) supplementation. Offspring neurodevelopment was assessed at postnatal days (PND) 5, 9 (**a**,**b** cliff aversion test; **c**,**d** surface righting test). Maternal supplementation with low dose fluoride (LF^−^) during pregnancy significantly improved pups’ neurodevelopmental outcomes when exposed to lipopolysaccharide (LPS)-induced intrauterine inflammation. This is shown by pups performance on the surface righting test at PND 9 compared to LPS groups alone. All groups with LPS exposure performed worse than control groups (phosphate buffered saline, PBS). One-way ANOVA with Bonferroni post hoc test, *p < 0.05, **p < 0.01. PBS, n = 7; LPS, n = 8; LPS + LF^−^, n = 9; PBS + LF^−^, n = 7.

A total of 31 litters were tested. Similar to the results of our previous studies, LPS-exposed pups displayed slower performance on neuromotor activity in cliff aversion and surface righting tests compared to PBS controls. Surface righting tests completed at PND9 show that maternal supplementation with LF^−^ significantly improved pups’ neuromotor behaviors compared to those in the LPS group (Fig. 3, p < 0.01, one-way ANOVA with Bonferroni post hoc test).

### Maternal LF^−^ supplementation prevented cortical neuronal injury after intrauterine LPS

**Figure 4:**
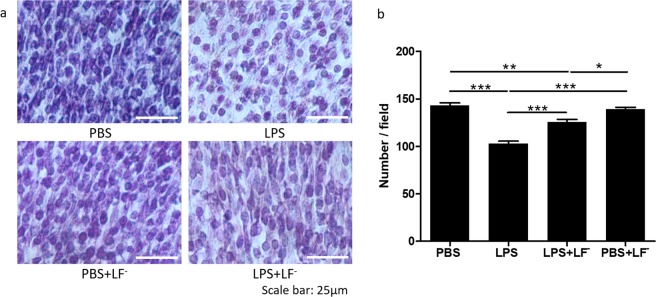
Cortical neuronal injury in offspring improved with maternal low dose fluoride (LF^−^) supplementation. (**a**) Routine Nissl staining was performed on fetal brains collected on embryonic day 18, 24 hours after lipopolysaccharide (LPS) exposure. Five fields were chosen at random from the frontal cortex, and neurons were quantified. (**b**) LPS exposure significantly reduced the number of neurons/field compared to phosphate-buffered saline (PBS) controls (***p < 0.001, one-way analysis of variance (ANOVA) with Bonferroni post hoc test). Fetal brains born to dams with LPS who were given LF^−^ supplementation during pregnancy had significantly more neurons than LPS alone (***p < 0.01, one-way ANOVA with Bonferroni post hoc test). Phosphate-buffered saline (PBS), n = 5; LPS, n = 5; LPS + LF^−^, n = 5; PBS + LF^−^, n = 5.

Neurons of E18 fetal brains were identified by Nissl staining, and changes in neuronal organization and number were assessed. In intrauterine LPS-exposed fetal brains, there were evidently dead neurons in the cortex, with rounded cell shapes and condensed nuclei (Fig. 4a). Nissl counting demonstrated that there was a significant decrease in the number of neurons in the LPS group compared to the PBS control group (p < 0.001, one-way ANOVA with Bonferroni post hoc test). Fetal brains from the group receiving maternal LF^−^ supplementation after LPS exposure had significantly more neurons than the LPS group alone (p < 0.01, one-way ANOVA with Bonferroni post hoc test) (Fig. 4b).

### LF^−^ treatment on human umbilical vein endothelial cells (HUVECs) increased cell viability and reduced oxidative stress after treatment with LPS

**Figure 5:**
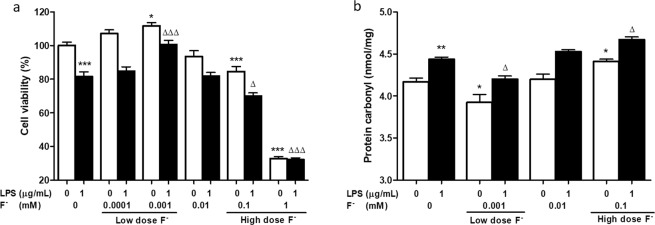
Low dose fluoride (LF^−^) treatment on human umbilical vein endothelial cells (HUVECs) increased cell viability and decreased oxidative stress after treatment with LPS. (**a**) LF^−^ treatment on HUVECs increased cell viability. HUVECs was incubated with the different doses of NaF for 6 h, and then LPS solution (1 µg/mL) was added to the culture wells for 18 h. The cell viability was determined using the CCK-8 assay. LF^−^ (0.001 mM) promoted HUVECs proliferation and high dose (HF^−^) fluoride (0.1 mM, 1 Mm) inhibited HUVECs proliferation. *p < 0.05, ***p < 0.001, one-way analysis of variance (ANOVA) with Bonferroni post hoc test, compared to control group (LPS 0 µg/mL and F^−^ 0 mM). ^Δ^p < 0.05, ^ΔΔ^p < 0.001, One way ANOVA with Bonferroni post hoc test, compared to LPS group (LPS 1 µg/mL and F^−^ 0 mM). (**b**) LF^−^ treatment on HUVECs reduced oxidative damage after treatment with LPS. Carbonyl protein level in the 0.001 mM F^−^ and LPS treated HUVECs group was significantly lower than that in LPS. In contrast, the HF^−^ group, which was treated with 0.1 mM F^−^ and LPS significantly higher than LPS groups. Data are expressed as mean ± SEM. *p < 0.05, **p < 0.01, one-way ANOVA with Bonferroni post hoc test, compared to control group. ^Δ^p < 0.05, One way ANOVA with Bonferroni post hoc test, compared to LPS group.

The effects of different fluoride concentrations on HUVECs viability were examined. LF^−^ (0.001 mM) promoted HUVECs proliferation, while HF^−^ (0.1 mM, 1 mM) inhibited HUVECs proliferation after treatment with LPS for 18 h (Fig. 5a). Protein oxidation is defined as the covalent modification of a protein induced directly by reactive oxygen species (ROS) or indirectly by a reaction with secondary by-products of oxidative stress^36^. Levels of oxidized proteins (protein carbonyl products) were significantly increased in HUVECs in the LPS group (LPS 1 µg/mL and F^−^ 0 mM) compared to those in the control group (LPS 0 µg/mL and F^−^ 0 mM) (p < 0.05, one-way ANOVA with Bonferroni post hoc test) (Fig. 5b). Carbonyl protein levels in HUVECs treated with 0.001 mM F^−^ and LPS were significantly lower than in HUVECs treated with just LPS (p < 0.05, one-way ANOVA with Bonferroni post hoc test). In contrast, HUVECs in the HF^−^ group, which were treated with 0.1 mM F^−^ and LPS, had significantly more carbonyl proteins than those in the LPS group (p < 0.05, one-way ANOVA with Bonferroni post hoc test).

## Discussion

For the first time, our study demonstrates that maternal low dose fluoride supplementation during IUI-affected pregnancy postpones the onset of PTB. It is clinically significant that the early PTB (Preterm delivery prior to 24 h) rate was lower in the LPS + LF group than in the LPS group before 24 hours. The gestation period in a mouse is about 20 days. Therefore, the 12-hour gestation period in a mouse represents more than one week in the gestation period in humans, when compared to a total gestation period of about 40 weeks. A one-week difference in the age of a premature baby is extremely important. As the age of preterm infants increases from week 22 to week 28 during the 28-week period, severe fetal morbidity and mortality decrease significantly^37^. Furthermore, low dose fluoride acts as an anti-inflammatory agent, to increase the liveborn rate and survival time of newborns following exposure to an intrauterine inflammatory environment. Moreover, maternal low dose fluoride supplementation prevents fetal cortical neuronal injury and results in neuromotor improvement following *in utero* inflammation exposure. The timing of administration and the dose of fluoride are very important, as the fluoride content increases in a dose- and time-dependent manner in all tissues and organs. High fluoride dosing has adverse effects, including increasing the PTB rate following LPS administration.

IUI is associated with PTB, with oxidative stress – an imbalance between the production of reactive oxygen species and defensive antioxidant mechanisms^38^ – playing a key role^39^. Basha, Rai and Begum found that the ingestion of high dose fluoride may lead to oxidative stress by increasing lipid peroxidation (which can greatly damage a cell) and modifying antioxidant enzymes^40^. We used a well-established mouse model of IUI and PTB^41^. In order to evaluate the translational potential of our study, we examined the direct effect of various doses of fluoride on HUVECs.

Many studies have used HUVECs to investigate the mechanism of preterm labor because dysfunctional endothelial cell activation and cytokines are implicated in preterm labor^42–44^. HUVECs are the most widespread *in vitro* model for the study of function and pathology of vasculature. The placenta represents the interface between maternal and fetal circulation, and features an extensive vascular network to facilitate nutrient provision from maternal blood, including fluoride. In our study, supplementation of low dose fluoride on HUVECs increased cell viability, and decreased oxidative stress.

More than 80% of human microbial infections – including gingivitis – have been associated with biofilms, which are clusters of microorganisms that exist in a liquid extracellular matrix^45^. Biofilms help evade the host immune response and are known to resist antimicrobial substances. Furthermore, some bacteria have been shown to resist antibacterial substances more in biofilms than under typical conditions, perhaps through affecting oxidative stress^45,46^. Biofilms are also important in PTB, as they can be present in amniotic fluid sludge, which is the presence of minute particles aggregated in amniotic fluid near the cervix. In human studies, the presence of amniotic fluid sludge has been identified as an independent predictor of imminent preterm delivery^47,48^.

Transient bacteremia (bacteria in the bloodstream)^49^ is known to be created during dental procedures, and can even be caused by routine toothbrushing^50^. We propose this as a possible mechanism for oral bacteria to access the placental membranes. Oral bacteria, including *Fusobacterium nucleatum* and *Porphyromonas gingivalis*, is known to be found in the placenta, and overall, the microbiome profile of the placenta is most similar to that of the oral cavity^21,51^. The use of intrauterine injection of LPS is used as an established model of PTB because it is an endotoxin present in the cell walls of gram-negative bacteria^52,53^. We propose that endotoxins of bacteremia from the oral cavity that are released at the placental membranes contribute to adverse pregnancy outcomes, including PTB. We used LPS from *Escherichia coli*, which is another gram-negative species, to serve as the oral bacterial endotoxin in our study. Thus, LPS is an effective model to imitate the effects of periodontal disease on pregnancy.

Prenatal supplementation of fluoride has been previously examined, yet solely in the context of caries prevention. Leverett *et al*. conducted a randomized trial, and found that more than 90% of the resultant children from both the treatment and control groups possessed no cavities. The researchers concluded that prenatal fluoride supplementation does not significantly reduce the risk of cavities in children^54^. Our study is the first to examine the effect of prenatal fluoride supplementation on PTB.

Prior research in rodents has shown that high doses of fluoride have detrimental effects when ingested during pregnancy, including accumulation in the fetal brain and a negative effect on cognitive function, such as learning, attention, and recollection^55,56^. Ge *et al*. showed that the offspring had a cognitive dysfunction in the group maternally exposed to high dose fluoride, and it was thought to most likely be caused by the disruption of the expression of synapse-associated proteins^57^. Similarly, our study demonstrates detrimental effects of high dose fluoride in PTB. Moreover, our research shows that low dose fluoride can potentially improve neurodevelopmental outcomes, through having an anti-inflammatory role, and a protective effect against cortical neuronal injury.

Our study is limited in its use of a mouse model, as more research must be performed in order to assess the effect of fluoride in the human pregnant population. Future clinical research studies have the potential to translate our findings in mice, possibly demonstrating fluoride’s ability to alleviate adverse pregnancy outcomes, such as PTB, that are associated with periodontal disease.

## Materials and Methods

### Mouse resource and regulation

All animal care and treatment procedures were approved by the Animal Care and Use Committee of Johns Hopkins University (Hopkins-IACUC Protocol No. MO14M326) and were carried out in accordance with institutional standards. Timed-pregnant CD-1® mice (Charles River Laboratories, Wilmington, MA, USA) were used in the study.

### Mouse model and group

On E17, an established mouse model of IUI was utilized as previously described^31–35^. Briefly, dams received 25 μg LPS (Sigma-Aldrich, St. Louis, MO, USA from Escherichia coli O55:B5) in 100 μL PBS or 100 μL PBS vehicle via intrauterine injection between the first and second gestational sacs of the right uterine horn. Dams were randomly assigned to 6 groups: PBS (n = 20), PBS + LF^−^ (n = 17), PBS + HF^−^ (n = 7), LPS (n = 52), LPS + LF^−^ (n = 51) and LPS + HF^−^ (n = 12). Mouse gestation lasts 19–20 days, and we consider all deliveries prior to E19 as preterm. PTB rates were evaluated at 24 h and 36 h after LPS injection. Pup survival data and delivery outcomes were assessed. Birth time was measured in hours relative to the time of LPS injection. Live pups’ birth weights were measured.

### Fluoride treatment

Based on NAM recommendations, pregnant women may receive 3 mg of supplemental fluoride each day to ensure adequate intake. Considering that women each day should drink a little more than 2 L of water (~1 mg fluoride/1 L tap water in Baltimore City)^58,59^ and brush their teeth twice (~0.25 mg fluoride/pea size amount of toothpaste)^60^ each day, they may consume up to 2.6 additional mg of fluoride daily. Thus, the maximum daily fluoride intake is approximately 5.6 mg, which is within the NAM and FDA limits. Considering a pregnant woman with an estimated weight of 80 kg, 5.6 mg/80 kg = 0.07 mg/kg/day. According to the FDA’s *Guidance for Industry: Estimating the Maximum Safe Starting Dose in Initial Clinical Trials for Therapeutics in Adult Healthy Volunteers*, the conversion equation for human and animal doses is as follows: Human mg/kg = Animal mg/kg dose × (Animal K_m_ ÷ Human K_m_); Human K_m_ is 37, and Mouse K_m_ is 3. The equivalent mouse fluoride dose equals 0.863 mg/kg/day. Assuming 0.04 kg/mouse, the total fluoride intake is 0.035 mg/mouse/day. According to guidance from the Johns Hopkins University Animal Care and Use Committee (http://web.jhu.edu/animalcare/procedures/mouse.html), average daily water intake for CD1 mice is 6 mL (1.5 mL/10 g body weight/day). Thus, mice received a low dose fluoride concentration of about 6 mg/L. Sodium fluoride (NaF) was purchased from Sigma-Aldrich (St. Louis, MO, USA). Timed-pregnant CD1 mice consumed water with 6 mg/L LF^−^ and 113 mg/L HF^−^ from E9 to postnatal day (PND) 19. Maternal water and food intake were recorded daily until delivery.

### Determination of fluoride concentration

After intake of low dose fluoridated water from E9 to E18, dams of each group were sacrificed on E18, and samples of serum and amniotic fluid were collected. Maternal urine was collected on E18 according to the previously described protocol^61^. Samples were directly analyzed for fluoride using a combination fluoride ion selective electrode (perfectION^TM^ Combination Fluoride Electrode, Mettler-Toledo, Columbus, OH, USA) and SevenExcellence pH/Ion meter S500 (Mettler-Toledo, Columbus, OH, USA). Samples were mixed 1:1 with Total Ionic Strength Adjustment Buffer II (TISAB II, Mettler-Toledo, Columbus, OH, USA) and placed under the electrode. The fluoride content of each sample (mg/L) was determined from a standard curve prepared by analyses of a series of fluoride standard solutions conducted at the same time.

### Behavioral evaluation

Tests were performed at PND 5 and 9 to assess the impact of LPS exposure and LF^−^ treatment on neuromotor and cognitive development in offspring. Cliff aversion and surface righting tests were performed according to the previously described protocol^62^. The amount of time needed to complete each test was recorded and analyzed. A total of 31 litters (PBS, n = 7; LPS, n = 8; LPS + LF^−^, n = 9; PBS + LF^−^, n = 7) were analyzed.

### Histochemistry of fetal brains

On E18, dams were sacrificed 24 h after surgery. Fetal brains were isolated and fixed in 4% paraformaldehyde overnight at 4 °C. Fixed tissues were immersed in 30% sucrose until saturated; they were then cryosectioned (20 μm thickness), followed by histological staining. Routine Nissl staining was performed to evaluate the cortical neuronal morphology in fetal brains. All photographs used for quantification were taken with Zeiss AxioPlan 2 Microscope System (Jena, Germany) attached to a Canon EOS Rebel Camera (Tokyo, Japan). Neurons were counted (field of view) based on Nissl staining using Image J (v1.48, http://imagej.nih.gov/ij/, National Institute of Health, Bethesda, MD, USA) on five randomly chosen fields in the frontal cortex.

### Cell culture and treatment

Human umbilical vein endothelial cells (201p-75n, Cell Applications Inc., San Diego, CA, USA) purchased in proliferating flasks were grown in Endothelial Cell Growth Medium (211–500, Cell Applications Inc, San Diego, CA, USA) in a 37 °C 5% CO_2_ humidified incubator. The growth medium was changed every 2 days. HUVECs were sun-cultured using Trypsin/EDTA Solution (070–100, Cell Applications Inc, San Diego, CA, USA) to make cells trypsinized, and Trypsin Neutralizing Solution (080-100, Cell Applications Inc, San Diego, CA, USA) was used to inhibit further tryptic activity. We used the HUVECs from passage 3 to passage 6. In this experiment, the HUVECs were treated with varying concentrations of NaF (0.0001, 0.001, 0.01, 0.1, or 1 mM). After incubation with NaF for 6 h, the LPS solution (1 µg/mL) was added to the HUVECs culture wells for 18 h.

### Cell viability assay and protein carbonyl measurement

HUVECs viability was determined with Cell Counting Kit-8 Assay Kit (CKK-8) (Dojindo Molecular Technologies, Inc., Rockville, Maryland, USA). The absorbance was measured with a microplate reader (BMG LABTECH, Inc., Cary, NC, USA) at a wavelength of 450 nm. Protein peroxidation was quantified as a marker of oxidative stress using the OxiSelect Protein Carbonyl ELISA Kit (Cell Biolabs, Inc., San Diego, CA, USA.).

### Statistical analyses

Statistical analyses were performed using Prism 6 (GraphPad Software, La Jolla, CA, USA). Categorical data were analyzed using either the Chi-squared test or Fisher’s exact test. Continuous data were tested for normality (Shapiro-Wilk normality test), and outliers were identified using Grubb’s test. Student’s t-test was used for parametric data. One-way analysis of variance (ANOVA) with Bonferonni post hoc testing was utilized for multiple comparisons of normally distributed data. Pups’ survival data and maternal oral intake data were analyzed by two-way ANOVA (group × time).

## Data Availability

The datasets generated during and/or analyzed during the current study are available from the corresponding author on reasonable request.
